# Supplementary material for: Polymorphisms in Pfkelch13 domains before and after the introduction of artemisinin-based combination therapy in Southwest Nigeria
Source: PLoS One. 2025 Mar 31;20(3):e0316479. doi: 10.1371/journal.pone.0316479 (PMC11957316; doi:10.1371/journal.pone.0316479)
Supplement: Supporting information 2 — (ZIP) [file pone.0316479.s002.zip › 029KNIFW_PREMIX_Plate_CORKELCH_E11.pdf]

Samples: 10322
Bases: 751
Average spacing: 14

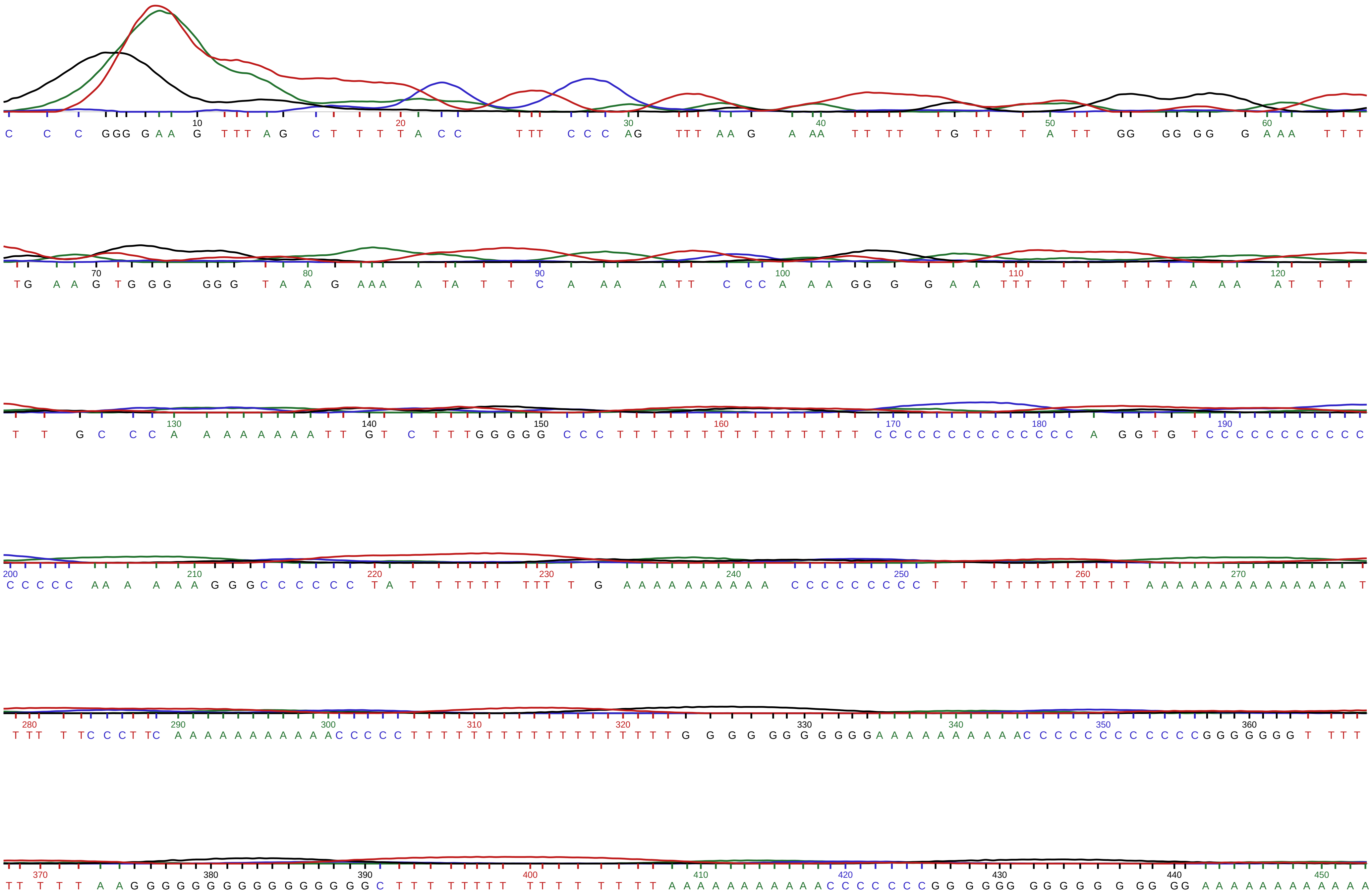

Samples: 10322
Bases: 751
Average spacing: 14

460 470 480 490 500 510 520 530 540

A C C C C C C C C C C C C T T T T T A A A A A A A A A A A A A A A A A A A A T T G T G T G G G G G G G G G G G G G G G G G G T T T T T T T T T T T T T T T T T T C C C C C G G

550 560 570 580 590 600 610 620

C G G G G A A A T T T T T T T T T T T T T T A T A A A A A A A A A A A A A A A C C C C C C C C C A C C G G C A A A G G G G G G A A G G A A A A A A A A A T T T T T T

630 640 650 660 670 680 690

T T T T T T T T T T T G T G G G G G G G G G G T G T G C C C C C C C C C C C C C C C C C C C C C A G A G A T A T G G T G G G A G A G G A A A

700 710 720 730

A T A T T A A T A T C T T C C T T G G C G G G A A A A G G C T T T T T T G A T G G

740 750

G G C C C C C C C C C C C C C
